# Exploring Plant Meiosis: Insights from the Kinetochore Perspective

**DOI:** 10.3390/cimb45100504

**Published:** 2023-09-28

**Authors:** Kang-Di Zhou, Cai-Xia Zhang, Fu-Rong Niu, Hao-Chen Bai, Dan-Dan Wu, Jia-Cheng Deng, Hong-Yuan Qian, Yun-Lei Jiang, Wei Ma

**Affiliations:** 1 Science Center for Future Foods, Jiangnan University, 1800 Lihu Road, Wuxi 214122, China; 17637083887@163.com (K.-D.Z.); zhangcaixia8445@126.com (C.-X.Z.); 2 School of Biotechnology, Jiangnan University, 1800 Lihu Road, Wuxi 214122, China; 1022200109@stu.jiangnan.edu.cn (H.-C.B.); 1023200332@stujiangnan.edu.cn (J.-C.D.); 1023210122@stujiangnan.edu.cn (H.-Y.Q.); 1023210125@stu.jiangnan.edu.cn (Y.-L.J.); 3 College of Forestry, Gansu Agricultural University, Lanzhou 730070, China; niufurong@gmail.com; 4 State Key Laboratory of Crop Gene Exploration and Utilization in Southwest China, Sichuan Agricultural University, Chengdu 611130, China; wudandan@sicau.edu.cn

**Keywords:** kinetochore, meiosis, spindle assembly checkpoint, chromosomal passenger complex, cohesin

## Abstract

The central player for chromosome segregation in both mitosis and meiosis is the macromolecular kinetochore structure, which is assembled by >100 structural and regulatory proteins on centromere DNA. Kinetochores play a crucial role in cell division by connecting chromosomal DNA and microtubule polymers. This connection helps in the proper segregation and alignment of chromosomes. Additionally, kinetochores can act as a signaling hub, regulating the start of anaphase through the spindle assembly checkpoint, and controlling the movement of chromosomes during anaphase. However, the role of various kinetochore proteins in plant meiosis has only been recently elucidated, and these proteins differ in their functionality from those found in animals. In this review, our current knowledge of the functioning of plant kinetochore proteins in meiosis will be summarized. In addition, the functional similarities and differences of core kinetochore proteins in meiosis between plants and other species are discussed, and the potential applications of manipulating certain kinetochore genes in meiosis for breeding purposes are explored.

## 1. Introduction

The macromolecular kinetochore structure, which is assembled by more than 100 structural and regulatory proteins on centromere DNA [reviewed in [1]], is the key player for chromosome segregation in both mitosis and meiosis. During cell division, kinetochores bridge the chromosomal DNA and microtubule polymers to mediate chromosome segregation and alignment [2]. In addition, they can function as a signaling hub to regulate anaphase onset via the spindle assembly checkpoint and control anaphase chromosome movement [2].

**Figure 1:**
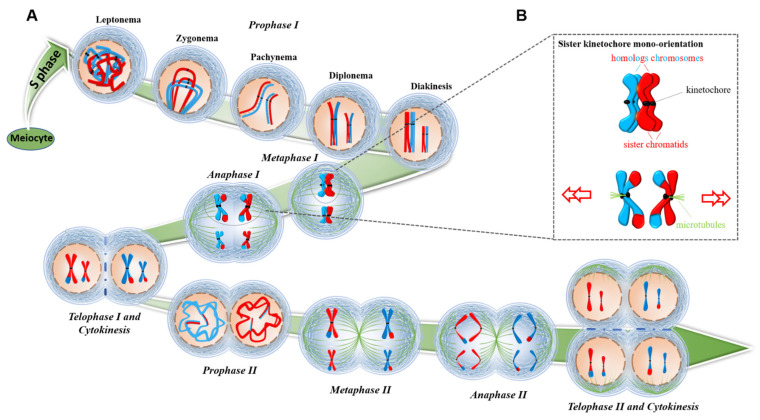
Overview of meiosis. (**A**). Chromosome segregation at different meiotic stages. At leptonema, chromosome axes are formed, and recombination is initiated. At zygonema, the synaptonemal complex is polymerized, where synapsis occurs and recombination proceeds. At pachynema, synapsis is completed, and recombination further progresses. At diplonema, the synaptonemal complexes are disassembled. Homologous chromosomes are connected by chiasmata. At diakinesis, chromosome condensation occurs, and bivalents can be distinguished. After prophase I, nuclear envelopes break down. At metaphase I, bivalents are aligned on the metaphase plate. At anaphase I, the release of arm sister chromatid cohesion allows the migration of chromosomes to two poles. Pericentromeric cohesion is specifically protected. At telophase I and cytokinesis, two nuclei form and chromosomes briefly decondense. In monocotyledons, cytokinesis occurs before meiosis II starts; in dicotyledons, cytokinesis happens only at telophase II. At metaphase II, sister chromatids are aligned on two metaphase plates. At anaphase II, sister chromatids separate following centromeric cohesion release. At telophase II, four nuclei are formed. At cytokinesis, haploid spores are released. (**B**). The panel shows the chromosome state at metaphase I and anaphase I (rectangle) magnified. Cohesion protection and sister kinetochore fusion occur during metaphase I to ensure the sister chromatids exhibit mono-orientation during anaphase I. For simplicity, only two pairs of homologous chromosomes with different lengths are shown. Each homologous chromosome has two chromosomes (blue and red) which distinguish the different parental origins. The kinetochore is depicted as a black sphere; the transiently accumulating synaptonemal complex is shown in green in prophase I stage; and the spindle is shown in green in other stages. Progression through different meiotic stages is denoted by a green arrow.

Meiosis is a special cell division type in which germ cells can produce gametes in the sexual reproduction stage among eukaryotes. A single round of DNA replication is followed by two sequential rounds of chromosome segregation, termed meiosis I and II (MI and MII). Accurate chromosome segregation during meiosis is fundamental for genetic material stable distribution. In plants, two types of meiotic divisions have been found. In plants with one single size-restricted centromere per chromosome (monocentric chromosomes), homologous chromosomes segregate during meiosis I, and sister chromatids separate in meiosis II (Figure 1). However, unlike monocentric chromosomes, plants such as *Luzula elegans* [3] and *Rhynchospora* [4] harbor holocentric chromosomes which lack a primary constriction site and form holokinetic kinetochores. The kinetochores are distributed along almost the entire poleward surface of the chromatids, to which spindle fibers attach. Due to this special chromosome structure, sister chromatids separate in meiosis I, whereas homologous chromosomes segregate in the second meiotic division in *Luzula elegans* [3] and *Rhynchospora* [4]. 

Understanding the mechanism of meiosis is critical since the mis-segregation of chromosomes leads to aneuploidy and chromosome instability and is the main cause for miscarriage, birth defects, and infertility in animals. In plants, abnormal chromosome segregation in meiosis can generate aneuploids or cause sterility [5,6]. However, the functions of many kinetochore proteins in plant meiosis have only recently been understood, and these proteins differ functionally from those found in animals.

In this review, we will summarize our current knowledge of how plant kinetochore proteins function in meiosis. In addition, the functional similarities and differences of core kinetochore proteins in meiosis between plants and other species are discussed. Additionally, potential applications for crop breeding practice by modifying some kinetochore genes in meiosis have emerged [5,6,7,8,9].

## 2. Kinetochore Structural Proteins

**Figure 2:**
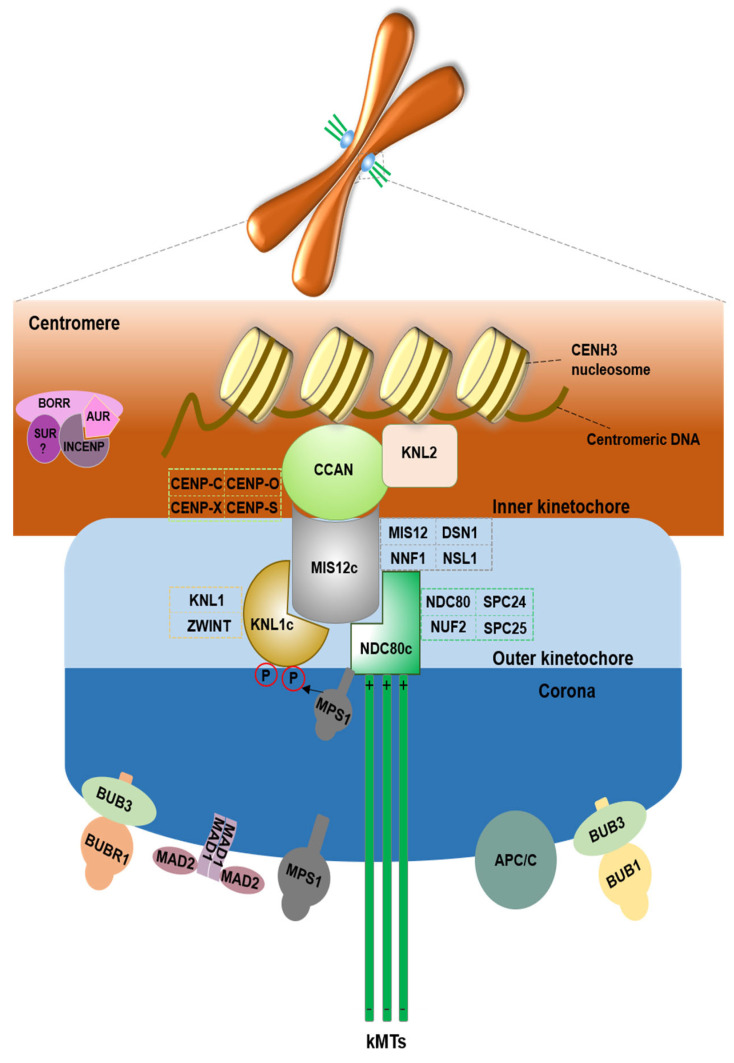
The centromere-kinetochore region.

Assembled by more than 100 structural and regulatory proteins on centromere DNA in eukaryotes [reviewed in [9]], the kinetochore is typically divided into two parts: the inner (DNA-proximal) and outer (microtubule-proximal) kinetochore [6] (Figure 2). Here, we will summarize how kinetochore structural proteins function in plant meiosis.

### 2.1. CENH3 Protein

**Figure 3:**
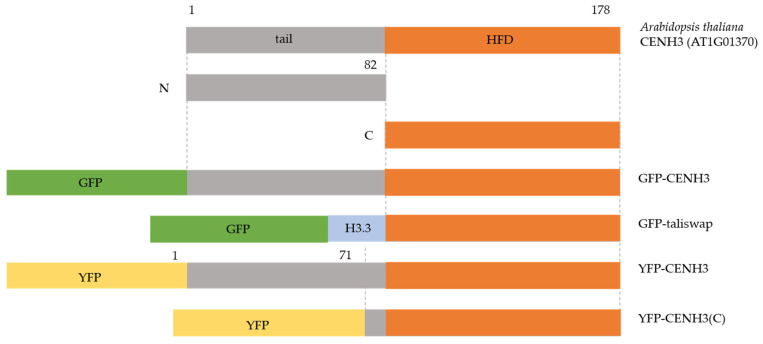
The schematic diagram illustrates the constructs utilized for expressing different CENH3 variants in *Arabidopsis* [13,15].

Plant centromeres are determined epigenetically by a specific histone H3 variant, called centromere histone 3 CENH3 (first described as human CENP-A [10]). Like other histone subunits, CENH3 carries an N-terminal tail (which protrudes from the nucleosome and is a target for posttranslational modification) and C-terminal Histone Fold Domain (which interacts with DNA and other histones from the nucleosome) (Figure 3). Contrary to conventional histones, CENH3 is evolving rapidly. The N-terminal tail of CENH3 is barely alignable even among closely related species, but the C-terminal Histone Fold Domain is largely conserved [reviewed in [11]]. The C-terminal part of CENH3 seems to be sufficient for mitotic centromere function in plants, but meiotic centromeres neither load nor tolerate impaired CENH3 molecules [12,13]. Ravi and Chan made the construct in which the hypervariable N-terminal tail of CENH3 was swapped out for the tail of the Schematic of the kinetochore showing simplified protein–protein interactions between major kinetochore components in plants. This illustrates the trilaminar structure of the kinetochore, as observed through electron microscopy. From inside to outside, the kinetochore’s structure can be broken down into three layers: the centromere (brown), the inner kinetochore (gray), the outer kinetochore (blue) and the kinetochore’s outer region, which is known as the fibrous corona (dark blue) [14]. A particular histone H3 variant (CENH3) specifies the position of the kinetochore in the centromere region. Various inner kinetochore parts throughout the cell cycle and connect with kinetochores: CCAN complex (which includes CENP-C, CENP-O, CENP-S, and CENP-X) and KNL2 protein. Particularly during cell division, numerous additional proteins are attracted to the outer kinetochore, including those in multiprotein complexes containing NDC80 (NDC80 complex, including Ndc80-Nuf2-Spc24-Spc25), MIS12 (MIS12 complex, including Mis12-Dsn1-Nnf1-Nsl1), and KNL1 (KNL1 complex, including Knl1 and ZWINT). They offer the spindle assembly checkpoint (SAC) proteins a landing platform, such as MPS1. The NDC80 complex can recruit the MPS1 and seems to be directly involved in microtubule binding. AUR, BORR, SUR (“?” represents the homologs not identified in the plant yet), and INCENP are chromosomal passenger complex (CPC) components that preferentially populate the centromere area and control the integrity of microtubule–kinetochore attachments. The SAC components (MPS1, BUB1, BUB3, BUBR1, MAD1, MAD2) and APC/C can be recruited to the fibrous corona.

The conventional H3.3 variant (encoded by At1g13370) carried GFP-tagged variants to the N-terminal tail domain (GFP-tailswap) (Figure 3). The GFP-tailswap expressing *Arabidopsis* plants showed sterility due to defects during sporogenesis, and the CENH3 signal was reduced to an undetectable level in meiocytes [15]. Moreover, *Arabidopsis* plants expressing the C-terminal part of CENH3-fused tag (EYFP-), named EYFP-CENH3(C) (C-terminal of CENH3 containing loop1 region) construct (Figure 3), showed chromosome segregation defects, decreased fertility, and the impaired loading of the tag (YFP-) signal in meiosis [13]. Strikingly, EYFP-CENH3(C) expression can even reduce the amount of endogenous CENH3; the insufficient CENH3 loading leads to the formation of lagging chromosomes and micronuclei [13]. Therefore, in contrast to mitosis, the N-terminal tail of CENH3 plays a key role in different loading mechanisms of CENH3 during plant meiosis [13].

GFP-CENH3, GFP-tailswap, YFP-CENH3, and YFP-CENH3(C) transgenes are described in this review paper. Tail, N-terminal tail domain; HFD, C-terminal histone fold domain; H3.3, the tail of conventional H3.3 variant; GFP, green fluorescent protein; YFP, yellow fluorescent protein.

### 2.2. Inner Kinetochore

The basic step of kinetochore formation is the incorporation of the CENH3 into nucleosome centromere positions. CENH3 was thought to physically interact with the inner kinetochore Constitutive Centromere-Associated Network (CCAN, which provides a platform for the assembly of centromeres and has been identified as playing a crucial role in recruiting the outer kinetochore protein complex) in most eukaryotes [16]. In vertebrates, CCAN consists of 16 CENP-named proteins: CENP-C, CENP-H, CENP-I, CENP-K, CENP-L, CENP-M, CENP-N, CENP-O, CENP-P, CENP-Q, CENP-U, CENP-R, CENP-T, CENP-W, CENP-S, and CENP-X [reviewed in [17]]. However, 12 out of the 16 CCAN components in vertebrates cannot be identified by homology search techniques in plants [18]. Only CENP-C, CENP-O, CENP-S, and CENP-X have been identified in moss *Physcomitrella patens*, an emerging model system for plant cellular biology [18]. It is unknown whether the CCAN complex occurs in the plants because CENP-S, CENP-X, and CENP-O were proven not to localize to the kinetochores in *Physcomitrella patens* [18]. In fission yeast, CENP-S and CENP-X, which are the CCAN network’s components, add the function of kinetochore assembly. By working with the Fanconi anemia pathway’s Fanconi Anemia Core Complex (FANCM) DNA translocase, CENP-S and CENP-X might also be involved in DNA repair [19]. The CENP-S and CENP-X present in *Physcomitrella patens* may be related to DNA repair. What function a CENP-O homolog might play on its own is not at all clear, especially when it does not localize to the kinetochore. Moss *Physcomitrella patens*, maize and *Arabidopsis* have been shown to contain CENP-C, the only remaining kinetochore component having a vertebrate CCAN homolog. However, regarding its function in meiosis, a thorough functional investigation is currently lacking [6,20,21]. In fission yeast [22], *Drosophila* [23], mice [24], and fish [25], CENP-C functions not merely as a structural link between the centromere and the kinetochore but also as joining the processes of early prophase homolog synapsis (means the alignment of chromosomes along its length and results in synaptonemal complex formation) to late metaphase kinetochore assembly and signaling in meiosis [reviewed in [26]]. 

**Figure 4:**
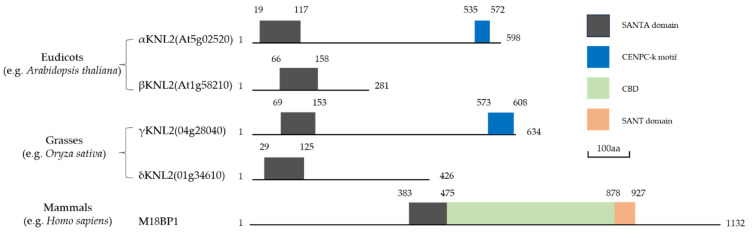
Schematic diagram of KNL2 conserved domain constructs in different species.

It has been found that in both animals and plants, CENH3 nucleosomes bind not only directly to CENP-C but also to a well-studied inner kinetochore protein KINETOCHORE NULL2 (KNL2, also called M18BP1 in vertebrates) [20,27,28,29,30]. KNL2 is responsible for the initiation of CENH3 deposition at the centromeres in humans [31], *C. elegans* [32], and fission yeast [31]. In plants, research has split KNL2 into eudicots αKNL2 (previous characterization) & βKNL2 (new representation) and grasses γKNL2 & δKNL2 (except that the maize genome contains only one copy of the δKNL2 gene) [33] (Figure 4). The conserved and characteristic Swi3-Ada2-NCoR-TFIIIB-associated (SANTA) domain has been found in all known KNL2 proteins. In some organisms, it is also accompanied by a putative SANT/ domain such as human [34] (Figure 4). Another conserved motif, named CENPC-like motif (CENPC-k), was identified on the C-terminal part of the KNL2 homologs in a wide spectrum of eukaryotes. However, human KNL2 does not contain a CENPC motif. A CENP-C binding domain (CBD), located in the middle part of the human protein, is required for the centromere localization instead [35,36] (Figure 4). In plants, research has found that *Arabidopsis* KNL2 can still target centromeres and interact with DNA even if the SANTA domain is deleted [29,30], likely because *Arabidopsis* KNL2 recognizes centromeric nucleosomes by the CENPC-k motif at its C terminus, which is required for KNL2 centromeric localization, similar to CENP-C [29]. All KNL2 act in both mitosis and meiosis [33]. Depletion of KNL2 in different organisms causes defects in CENH3 assembly. Knockout of M18BP1 in human HeLa cells with RNAi abolished centromeric recruitment of newly synthesized CENP-A, leading to chromosome mis-segregation and interphase micronuclei [37]. In *Arabidopsis*, knockout of αKNL2 via a T-DNA insertion showed reduced CENH3 loading at the centromeres and chromosome segregation defects in both mitosis and meiosis [30]. Conversely, αKNL2 expression is stable in CENH3-RNAi transformants, indicating that αKNL2 acts upstream of CENH3 and has a function in the assembly of CENH3 at the centromeres [30]. Moreover, βKNL2 knockout leads to cell cycle disorder, such as abnormal seed development, and a semi-lethal mutation phenotype indicating the defects in meiotic and mitotic chromosome segregation in *Arabidopsis* [33].

The figure shows the KNL2 conserved domain of eudicots (*Arabidopsis thaliana)*, grasses (*Oryza sativa*), and mammals (*Homo sapiens*). There are two KNL2 proteins in *Arabidopsis thaliana*, αKNL2 & βKNL2, and two KNL2 proteins in *Oryza sativa*, γKNL2 & δKNL2. However, the *Homo sapiens* category only has one KNL2—M18BP1. SANTA domain, CENPC-K motif, CBD domain, and SANT domain are all highlighted in different colors: black for the SANTA domain, blue for the CENPC-K motif, cyan for the CBD, and brown for the SANT domain. The graphic demonstrates that KNL2 and KNL2 have comparable domains. The figure shows that the domains of αKNL2 and γKNL2 and those of βKNL2 and δKNL2 are comparable.

### 2.3. Outer Kinetochore and KMN Network

An outer kinetochore subcomplex called the KMN network (KNL1-MIS12-NDC80) that binds to spindle microtubules is recruited by the CCAN network in the inner kinetochores, which also connects with the centromere [38,39,40,41]. The KMN complex includes: KNL1c (consisting of Knl1 and ZWINT), NDC80c (consisting of Ndc80-Nuf2-Spc24-Spc25 proteins), and MIS12c (consisting of Mis12-Dsn1-Nnf1-Nsl1 proteins). MIS12c anchors NDC80c and KNL1c from yeast to humans (Figure 2) [40,41,42,43,44,45,46,47]. Like centromeric proteins, most kinetochore proteins are fast evolving and, hence, display less sequence homology between their respective orthologs despite their functional conservation. Only a few kinetochore proteins from the KMN group that offers high sequence identity with the yeast or mammalian counterparts have been characterized in plants. In *Arabidopsis*, the interaction of necessary for nuclear function 1 (NNF1) and MIS12 has been confirmed in Y2H and co-immunoprecipitation tests [45]. Shin et al. (2018) confirmed that AtNUF2 directly interacted with AtNDC80 and AtSPC25 subunits in *Arabidopsis* by the yeast two-hybrid analysis. Recently, studies of the NDC80 complex in *Arabidopsis* showed that MERISTEM UNSTRUCTURED (SPC24/MUN) interacts conservatively with subunits NDC80, NUF2, and SPC25 using co-immunoprecipitation analysis, and MUN protein has a coiled-coil region and a globular domain at the end, which are typical structural features found in all four components of the NDC80 kinetochore complex [47].

In the KMN group, only NDC80 [40], MIS12 [41], NNF1 [45], NUF2 [46], and SPC24/MUN [47] homologs are functionally characterized in plants. They display conserved functions in chromosome segregation and microtubule organization during cell division. In maize, the localization patterns of Knl1 signals overlap with those of Mis12 and Ndc80, indicating that the deficiency of Knl1 can likely impair kinetochore function. This impairment leads to abnormal chromosome behavior during cell division in early endosperm development, ultimately resulting in defective kernels [38]. During meiosis, maize MIS12 interacts with NDC80, forming a visible MIS12-NDC80 bridge. This bridge fuses sister kinetochores, directing sister kinetochores’ mono-orientation behavior during metaphase I and initiating the homolog chromosomes segregation [41]. After systemically reducing the levels of MIS12, the MIS12-NDC80 bridge is broken. This leads to kinetochores orienting and separating randomly in metaphase I, causing chromosome non-disjunction in anaphase I due to the presence of sister chromatid cohesion [41]. Another component of the MIS12 complex, NNF1, *atnnf1-1* mutation in *Arabidopsis* causes embryo mortality, which shows its crucial significance in kinetochore function during cell division [45]. For the NDC80 complex, it has been shown that the mutation of AtNUF2 led to severe mitotic defects, not only in the embryo and endosperm but also in seedlings, resulting in seed abortion and stagnating seedling growth [46]. Moreover, the partially complemented *nuf2-3/-^DD45;ABI3pro::AtNUF2^* (*nuf2-3/-^DA^*) root apical meristem cells, along with the aberration of spindle MTs, resulted in blocked root growth [46]. Furthermore, AtSPC25 could co-localize with CENH3 and MT arrays in various phases of mitosis suggesting that the AtSPC25 gene may perform a similar function as AtNUF2 [46]. Recently, studies of another component of the NDC80 complex in *Arabidopsis* showed that the *mun-1* mutant, which is a weak allele because of the insertion of T-DNA in the promoter region of the SPC24 homolog, displays stunted growth, embryo arrest, DNA aneuploidy, and problems in chromosome segregation with a low cell division rate [47]. Additionally, Null mutants of MUN from TALEN and CRISPR/Cas9-mediated mutagenesis demonstrated zygotic embryonic lethality comparable to *nuf2-1*, indicating it is necessary for proper cell division [47].

## 3. Spindle Assembly Checkpoint (SAC) Proteins

During cell division, the spindle assembly checkpoint (SAC) acts to maintain genome stability by prolonging metaphase “stagnation” until all kinetochores correctly attach to the microtubule spindle apparatus. Once all kinetochores become stably attached to spindle, the SAC is inactive, which allows chromosome segregation and cell division to proceed. The SAC core proteins, including monopolar spindle 1 (MPS1), budding uninhibited by benomyl (BUB: BUB1, BUB3), BUB1-related protein 1 (BUBR1, also called MAD3), and mitotic arrest deficient (MAD: MAD1, MAD2) have been identified in divergent branches of the eukaryotic kingdom, including yeast, animals, and plants (Table 1); for reviews see [48,49]. Therefore, it seems that the SAC appears to be an ancient control mechanism [50]. In mitosis and meiosis, the regulatory mechanisms of the SAC proteins and the relationship between them have all been well studied [51,52,53]. Here, we will focus on the plant SAC proteins for their mechanisms during meiosis. 

### 3.1. MPS1 Protein

MPS1 protein was originally found in yeast and identified as a kinase [65,66]. MPS1 orthologues can be identified in all supergroups of eukaryotes and in metazoan, with the exception of nematodes [67]. MPS1 protein as an upstream SAC regulator initiates SAC signaling, which has been proven in yeast [68] and human cells [69,70,71,72]. MPS1 phosphorylates Spc105/KNL1 at several phosphorylation sites to start a biochemical cascade that recruits almost all other SAC components directly or indirectly to kinetochores and monitors accurate chromosome alignment [69,73,74,75,76]. In recent years, the function of MPS1 protein in plant meiosis has been released. Like yeast [77], *Drosophila* [78,79], and mice [80], a mutation of MPS1 leads to chromosome mis-segregation and aneuploidy because of the precocious entry into anaphase I with erroneous kinetochore attachments in *Arabidopsis*, which finally leads to male and female gametophyte abortion [54]. Moreover, MPS1(AtPRD2) is involved in the formation of double strand break (DSB) and spindle structure during meiosis in *Arabidopsis* [55]. However, the rice OsPRD2 does not participate in spindle assembly during meiosis I but shows a conservative role in the meiotic DSB generation [56,57]. Consistent with other DSB defective mutants, later synapsis and recombination were disrupted in *osprd2* [57]. This indicates that the function of MPS1(PRD2) is different among plant genomes of different lineages; further studies need to be explored in different plants.

### 3.2. BUB Proteins

BUB proteins include BUB1 kinase (also called BMF1 in plants), BUB3, and an unusual pseudo-kinase BUB1-related protein1 (BUBR1, also called MAD3). In yeast and mammals, BUB1 localizes to kinetochores through the BUB3-binding domain. Although BUB1 homologs in plants have also been found, a BUB3-binding region named Gle2-binding-sequence (GLEBS) domain is missing in all plant BUB1 proteins [58,81,82]. In fission yeast, BUB1 is required for localization and the centromeric protection of cohesin subunit Rec8 during meiosis I, which may be because of the interaction between BUB1 and Shugoshin (Sgo) [59,83,84]. It has been shown that the N-terminus of BUB1 is necessary for the targeting of Sgo1 to centromeres and the protection of cohesion, whereas the C-terminal acts together with Sgo2 to promote sister kinetochore co-orientation at the MI stage during fission yeast meiosis [59].

In plants, rice *brk1* (homologs of BUB1 in rice and located at the kinetochore) display a sterile phenotype because of the precocious loss of sister chromatid cohesion at the onset of anaphase I. Then, the tension between homologous kinetochore reduces, which finally leads to a sterile tetrad phenotype [58]. In maize, the ZmBub1 RNAi transgenic line also showed a decline of anther fertility to certain degrees [82]. Moreover, maize ZmBub1 can localize to metaphase I centromeric regions in *sgo1* mutants [82], which suggests that ZmBub1 localization to kinetochores is independent of ZmSgo1 during maize meiosis. Furthermore, it has been shown that in maize *afd1* (homolog of Rec8), mutant ZmBub1 is detectable during the entire meiotic division. Taken together, ZmBub1 recruitment is Rec8- and Sgo1-independent [82]. However, *Arabidopsis bub1* mutants are morphologically similar to wild-type even under microtubule-destabilizing conditions [81]. 

The roles of Bub3 have been well studied in mouse oocytes, and similarity between mammalian mitosis and meiosis has been illustrated [85]. Overexpression of Bub3 causes meiotic arrest while depletion of Bub3 from kinetochores causes chromosome misalignment and abnormal polar body extrusion, leading to aneuploidy [85]. In plants, three homologs of BUB3: BUB3.1, BUB3.2, and BUB3.3 have been identified in *Arabidopsis*, and they all contain WD40 repeats which are required for mitotic checkpoint complex formation [86,87]. BUB3.1 and BUB3.2 show 88% sequence similarity, and BUB3.3 only shares 37% amino acid identity with BUB3.1 and BUB3.2 [87]. In *Arabidopsis*, the localization pattern of BUB3.1:GFP and BUB3.2:GFP are present at the kinetochores in mitotic cells, BUB3.3:GFP is present in the cytoplasm, indicating a plant specific accumulation pattern that does not correspond to established SAC function in animal and yeast cells [88]. In *Arabidopsis*, loss of BUB3.1 function leads to embryonic lethality, *bub3.2* mutants do not exhibit any defects, and there is missing information about *bub3.3* mutants [87,88,89]. In maize meiosis, BUB3 signals were determined by immunostaining, which appeared first as weak signals in interphase and then became stronger through prophase I. The signals persisted at the kinetochores throughout meiosis I and II [82]. 

MAD3/BUBR1 is a paralogous protein of BUB1 and contains a MAD3/BUB1 domain, a GLEBS domain, and an inactive pseudo-kinase domain. MAD3/BUBR1 has been identified in yeast [90], *Drosophila* [90,91], a mouse [92], and plants [93]. In a mouse [92,94] and mice [95], BubR1 depletion by RNAi accelerated meiotic progression, and overexpression of BubR1 caused meiotic arrest. Moreover, cold treatment disrupted spindle microtubules in BubR1-depleted oocytes, suggesting that BubR1 monitors kinetochore–microtubule attachments [92]. Furthermore, BubR1 is essential for maintaining sister chromatid cohesion during meiotic progression in both sexes in *Drosophila* [96]. Two MAD3/BUBR1 homologs (called MAD3.1 and MAD3.2) have been found in *Arabidopsis* and maize; they all lost their GLEBS domain and pseudo-kinase domain (Review in [93]). MAD3.1 (also called BMF2 in plants) localizes to kinetochore under microtubule-destabilizing conditions. In addition, in MAD3.1 and MAD3.2 (also called BMF3 in plants), direct interaction was observed at kinetochores, indicating that the interaction only occurs when the SAC is active [97]. However, their function in meiosis is still unclear in plants. 

### 3.3. MAD Proteins

MAD proteins, including MAD1 and MAD2, have been identified in almost all eukaryotes. The kinetochore localization of MAD1 has not been reported, but MAD2 homologs have been shown to strongly accumulate at kinetochores under microtubule-destabilizing conditions in *Arabidopsis*, maize, and wheat [97,98]. Under SAC-activated conditions, MAD1 localizes predominantly to unattached kinetochores and recruits MAD2 to form the MAD1–MAD2 complex. MAD1 is a coiled-coil protein with a MAD2 interaction motif; the MAD1–MAD2 interaction has been confirmed by gel filtration and co-immunoprecipitation experiments in budding yeast [99]. The loss of MAD1 causes chromosome dislocation and premature entry into anaphase I in yeast [100], *Caenorhabditis elegans* [101], and a mouse [102]. In *Arabidopsis*, localization of MAD1 to the nuclear periphery was also observed, but the relationship between SAC components and DNA repair has not been studied yet [103]. Interestingly, it has been shown that *Arabidopsis* MAD1 regulates flowering time and endo-polyploidization, suggesting that MAD1 is involved in cell cycle control in the timing of reproductive transition [104].

MAD2 was reported to localize to kinetochores during meiosis in a mouse, a rat, maize, and a grasshopper [60,105,106,107]. However, the patterns of MAD2 dynamics are different among species. Specifically, in meiosis I of maize, MAD2 localizes to unattached kinetochores. After proper attachment, it is lost from the kinetochores, indicating that MAD2 may sense microtubule attachment [107]. However, in mouse spermatogenesis, MAD2 is shown to remain at most kinetochores throughout meiosis I and is lost only during metaphase of meiosis II. The persistence of MAD2 at kinetochores in oocytes was seen during meiosis II [105]. It has been shown that MAD2 is a key SAC protein involved in the regulation of meiotic chromosome segregation. In the absence of MAD2, the meiotic cells showed similar defects as MAD1 mutants during meiosis I in yeast and a mouse [108,109,110]. In maize, the loss of MAD2 staining in meiosis was not correlated with initial microtubule attachment but was correlated with a measure of tension: the distance between homologous or sister kinetochores (in meiosis I and II, respectively). After the staining of tension-sensitive 3F3/2 (the 3F/2 antibody recognizes a phosphor-epitope that is localized to prometaphase kinetochores until the chromosomes have aligned properly at the metaphase plate), phosphor-epitope was present and absent concomitantly with MAD2 at the meiotic kinetochores [107]. 

## 4. Chromosomal Passenger Complex (CPC) Proteins

Accurate chromosome segregation to avoid chromosomal instability and aneuploidy is guaranteed by the SAC. The loading and function of SAC at the kinetochores depend on a few complexes, especially the chromosome passenger complex (CPC). Thus far, core enzyme Aurora kinase and three non-enzymatic kinases including inner centromere protein (INCENP), Borealin, and Survivin in CPC proteins have been identified in most organisms (Table 1). CPC components are preferentially populated in the centromere area (reviewed in [111]), and CPC is known in correcting kinetochore–microtubule attachments [112,113], activating the SAC complex [112], stabilizing the spindle midzone [113,114], and promoting cytokinesis [115] during cell division. Aurora B is the core component in CPC. The N-terminus of auroraB kinase binds to the C-terminus of INCENP, while Survivin and Borealin bind the N-terminus of INCENP (Figure 2).

### 4.1. Aurora Kinase

The Aurora kinases are a family of highly conserved serine/threonine kinases. Yeasts, including *Saccharomyces cerevisiae* and *Schizosaccharomyces pombe*, contain a single Aurora homolog, Ipl1p (increase in ploidy 1) [116] and Ark1 (Aurora-related kinase 1, an Aurora-B-like kinase) [117], respectively. At least two functionally divergent Aurora members of the multicellular eukaryotes are present: Aurora A and Aurora B. Mammals can have up to three Aurora genes: Aurora A, B, and C [118]. In plants, three homologs of Aurora categorized into two groups were identified in *Arabidopsis* and rice: α-Aurora (AUR1 and AUR2, similar to Aurora A in mammals and locates to the spindle microtubule), β-Aurora (AUR3, similar to Aurora C in mammals and locates to the kinetochore) [61,62,119]. It has been shown that Aurora kinases play an important role in centrosome maturation, spindle assembly, meiotic maturation, and metaphase I spindle orientation [120], as well as regulate SAC activity [121], control the kinetochore orientation during meiosis [122], and function specifically in meiotic spindle attachment in oocytes [123] and during spermatogenesis [124].

It has been shown that all three AtAurora kinases localize to male and female gametophytes, suggesting a possible prominent function during plant meiosis. In contrast to yeast and animals, the role of plant Aurora kinases in meiosis is only starting to emerge. In *Arabidopsis*, the reduced activity of Aurora kinases (AtAurora1, −2, −3) leads to meiotic defects and the formation of unreduced pollen including plants with an increased ploidy level [125,126]. This not only reduces expression of Aurora kinases but also overexpression results in genomic instability [125]. In *Arabidopsis*, overexpression of any member of the AtAurora family is not possible (overexpression of untagged AtAurora kinases is detrimental for plant development), so only plants with tagged [GFP, tandem affinity purification (TAP) tag] or truncated forms of Aurora kinases can be expressed. This indicates that the activity of Aurora kinases is affected by the affinity tag, making their overexpression possible for plants [125]. *Arabidopsis* 35S::AtAurora1-TAP transformants showed increased endopolyploidization, disturbed meiosis, and the formation of small amounts of tetraploid seeds [125]. Overexpression of truncated AtAurora1 resulted in an imbalanced segregation of chromosomes during meiosis and the formation of 39% aneuploid seeds [125]. Thus, in plants, balanced expression of Aurora kinases is essential for the proper execution of mitotic and meiotic divisions, and therefore for overall growth and development [125,126,127]. Analysis of *aurora1 aurora2* double mutants and Ataurora3 RNAi transformants in the *aurora1* mutant background showed that they exhibited disturbed meiosis and form triploid or a combination of triploid and tetraploid seeds, respectively [125].

### 4.2. Other CPC Components

INCENP is the largest non-catalytic subunit of the CPC, which binds to all other CPC components directly in yeast and animals. The N-terminal region of INCENP interacts with Borealin and Survivin, while the C-terminal domain, called the IN-box, with four amino acid residues binds to Aurora B [128,129]. Like yeast and animals, a putative *Arabidopsis* ortholog of INCENP, named WYR, contains a characteristic C-terminal domain and a coiled-coil domain; an IN-box aurora B-binding domain has been identified as well. In addition, *Arabidopsis* WYR has a long N-terminal region of unknown function, which is only conserved in plants [130]. In general, INCENP functions in the CPC complex to regulate cell cycles, including chromosome segregation, the spindle assembly checkpoint, and cytokinesis [64]. However, in plants, it is still unclear if INCENP acts as a putative plant CPC because of missing information about its CPC binding partners. WYR has been found to play a role in meiosis; *Arabidopsis wyr-1/+* produces dyads and triads in male sporogenesis, indicating the failure of chromosome segregation (meiotic non-reduction) during meiosis [130]. 

N-terminal Borealin acts as the INCENP-binding region, while its C-terminal part consists of a homodimerization domain that is involved in stable CPC localization to centromeres [63]. The location of the CPC complex on the chromosomes depends on the phosphorylation status of the histones during mitosis and meiosis [131]. BOREALIN RELATED (BORR, Borealin homolog in *Arabidopsis*) has been identified in *Arabidopsis* and in most branches of the plant kingdom, which co-localizes with INCENP homologs to the central region of the kinetochore and the film-forming body during mitosis and meiosis [130]. BORR is required for proper chromosome segregation during cell division in *Arabidopsis* and is strongly expressed not only in proliferating cells but also in flowering tissues, including male and female reproductive organs [129]. *Borr* mutants showed lagging chromosomes and abnormal cell division, resulting in undeveloped ovules, aborted seeds, and embryonic defects [129], indicating its role in meiosis.

In yeast and animals, Aurora, INCENP, and Borealin, together with Survivin, form the CPC complex [111]. However, Survivin is still missing from the picture in plants, raising the question whether complete and functional canonical CPC exists in plants. In a recent report, Komaki, S. et al. identified *Arabidopsis* BOREALIN RELATED INTERACTOR 1 and 2 (BORI1 and BORI2) as redundant Survivin-like proteins in the context of the CPC in plants [132]. In rat and mouse oocytes, Survivin is a critical regulator of spindle assembly checkpoint activity and chromosome alignment during meiosis [133,134]. However, it is not yet clear whether BORI1 and BORI2 function in plant meiosis.

In total, little is yet known about the role of plant CPC complex, especially in meiosis. Further genetic and biochemical analysis is needed to confirm plant CPC components and to understand their roles in meiosis.

## 5. Centromeric Cohesion in Meiosis

To ensure that each gamete receives only half the number of parental chromosomes, meiocytes undergo a reductional division separating parental homologs during meiosis I. The kinetochores of sister chromatids fuse and then point toward the same direction (mono-orientation) in meiosis I (Figure 1), allowing homologous chromosomes to correctly segregate to different sides [135,136]. This is partly mediated by sister chromatid cohesion. The cohesin complex forms a ring-like structure that comprises four main subunits: structural maintenance of chromosomes 1 (SMC1) and SMC3 protein, and the α-kleisin SCC1 (also called RAD21 or REC8) and the SCC3 proteins [137,138,139,140,141]. Studies demonstrated that SCC1 is acting on the mitotic cohesin complex and is replaced during meiosis by REC8 in almost all eukaryotes [142,143,144]. 

### 5.1. Sister Chromatid Cohesion

In meiosis I, to make sure homologous chromosomes segregate, sister kinetochores of each homolog have to face toward the same spindle pole. Therefore, sister chromatid cohesion must be released in two steps: in meiosis I, loss of cohesion at the chromosome arm releases chiasmata and enables the reductional segregation [142,145]. Moreover, the cohesion in the centromeric region is retained until metaphase II, which ensures the segregation of sister chromatids during anaphase II [146]. Mutants of Rec8 lead to cohesion protection defect at the centromeric region, which has been proven in many organisms [135,146,147,148,149]. Moreover, SMC1 and SMC3 are the vital cohesin proteins during meiosis, and AtSMC3 exists in the cytoplasm and nucleus and the chromosome and nuclear matrix in *Arabidopsis* in meiosis and mitosis cells. During meiosis, at the prophase of meiosis, AtSMC3 was located along the sister chromatids to the axial and lateral elements, and at metaphase I, AtSMC3 was only located in the centromere [150]. Interestingly, tomato SMC1 and SMC3 show similar positioning modes to AtSMC3, but no spindle position was recorded in tomatoes [151]. This indicates that the function of plant cohesin subunits is consistent with other eukaryotic organisms in meiosis (a detailed review of plant cohesin in meiosis can be found in [127,140,152,153,154]).

### 5.2. Mono-Orientation

In the last two decades, several meiosis-specific proteins, including budding yeast Spo13 [155], fission yeast Moa1 [156], mouse MEIKIN [157], and *Drosophila* Mtrm [158], have been identified with crucial functions in mono-orientation. These proteins, so-called meiosis I kinase regulators (MOKIRs), mediate the segregation of homologous chromosomes (mono-orientation). The MOKIRs bear no obvious sequence similarity, although their functions are conserved. However, MOKIRs are missing from the picture in the plants, raising the intriguing possibility that MOKIRs have been lost in the green lineage and that the plant meiosis mono-orientation is regulated by different mechanisms. It has been suggested that kinetochore mono-orientation also depends on Rec8 in many species, including yeasts [159], *C. elegans* [160], *Drosophila* [161], and plants [135,162,163]. In plants, mutants of meiosis-specific α-kleisin Rec8 homologs, including *Arabidopsis* SYNAPSIS1(*syn1*--also called DETERMINATE INFERTILE1, *dif1*) [141,164,165], rice *osrad21-4* [163,166], and maize *afd1* (absence of first division) [167] exhibit an equational segregation of chromosomes at metaphase I. In addition to REC8, SCC3 is a desired protein for the mono-orientation during meiosis I [168]. Through a blast search of SCC proteins of many species, its homologous protein AtSCC3 was found in *Arabidopsis*, which involves the male and female meiosis, and its disruption causes the early release of cohesion at anaphase I [135,169].

### 5.3. Cohesin Protectors

Shugoshins (SGOs, which means ‘guardian spirit’ in Japanese) were firstly identified in *Drosophila* (MEI-S332) [170], and successively described in yeast, mammals, and plants [171]. A conserved feature of shugoshin proteins is their localization to pericentromeres during meiosis I, consistent with their role in protecting cohesion in this region [170,172,173,174,175,176,177,178]. Research in yeast and vertebrates has shown that SGO is phosphorylated by kinase Aurora B and binds as a homodimer with protein phosphatase 2A (PP2A). Then, the complex dephosphorylates the cohesion subunit REC8 to protect it from separase (cysteine protease) during meiosis [179,180,181,182]. There are differences in both amino acid sequences and certain accessory functions among SGOs from different species [183,184]. The organisms including fission yeast, plants, and mammals contain two shugoshin-like proteins (Sgo1 and Sgo2, acting differently in mitosis and meiosis I), in contrast to budding yeast and *Drosophila*, which only contain a single shugoshin protein Sgo1 [170,173]. Sgo1 is predominantly responsible for the protection of centromeric cohesion during meiosis in fission yeast and plants [173,176,183,185], whereas Sgo2 carries out this function in mammals [178]. The role of SGO has been identified in *Arabidopsis*, maize, and rice, and the *sgo1* mutants show the precocious separation of sister chromatids due to loss of centromeric cohesion at anaphase I, producing unbalanced chromosomes at MII [176,183,184,185,186]. Furthermore, in *Arabidopsis*, *atsgo1 atsgo2* double mutants showed that the frequency of unbalanced meiotic products had significantly increased compared with the single *atsgo1* mutant [176,184,185,186]. 

Moreover, a plant-specific protein PATRONUS1 (PANS1,) has been identified in *Arabidopsis* [184,185]. Similar to SGOs, PANS1 is not required for monopolar attachment of sister kinetochores in meiosis I. However, in contrast to the SGOs, *pans1* meiocytes show a premature release of sister chromatid cohesion at metaphase II but not at meiosis I, indicating that the protein is required for the protection of cohesion during interkinesis, at a later stage than SGOs [184,185]. PANS1 may be a regulator of the APC/C complex because of the interaction with some of the APC/C subunits revealed by TAP-TAG and Y2H experiments [184]. In rice and wheat, there are also PANS1 similar proteins identified, named RSS1 and TdRL1, respectively [187,188,189]. However, the mechanism of PANS1 is still unclear.

## 6. Concluding Remarks and Perspective

In plants, altered expression of key meiotic regulators at kinetochores results in impaired meiotic divisions followed by the formation of aneuploid or polyploid progenies. For example, manipulating Aurora kinase activity by Aurora inhibitors efficiently resulted in the formation of aneuploids and polyploids [125]. This observation is interesting from an evolutionary point of view because it can be assumed that, during evolution, the influence of biotic and abiotic factors on the activity of meiotic genes resulted in the formation of aneuploids or polyploids that served as a basis for the formation of new species. In addition, this knowledge can be used for applied research because manipulating key meiotic genes can efficiently result in apomixis and the induction of haploids. For example, inactivating a few key meiotic genes including Rec8 activity can induce apomixis into rice [3] and breed seedless watermelon varieties [7]. In addition, it has been demonstrated that modulation of CENH3 can introduce haploids in *Arabidopsis* [190,191] and in crop species such as maize [192], wheat [193], and barley [194]. Detailed studies in this direction might help in understanding the problem of plant genome stability. Compared with animal and yeast kingdoms, many of the meiotic studies regarding the kinetochore perspective in plants have just started. The presence of numerous plant-specific phenotypes in kinetochore meiotic regulator mutants, in contrast to the observed functional divergence between animal homologs, makes it intriguing to further unravel these key kinetochore meiotic regulators in meiosis.

## Figures and Tables

**Table 1:** List of SAC and CPC proteins in plant meiosis.

Name	Homologs	Protein Function or Feature	Mutant Phenotype	Reference
SAC proteins				
MPS1	AtMPS1 *(Arabidopsis)*	required for faithful chromosome segregation	chromosome mis-segregation; aneuploidy; precocious into anaphase I	[54]
	AtPRD2 (*Arabidopsis*)	involves the formation of DSB and spindle structure	gametophytes aborted; abnormal meiosis products	[55]
	OsPRD2 (Rice)	meiotic DSB formation	male and female completely sterile; twenty-four univalent	[56,57]
BUB1	BRK1 (Rice)	proper tension between homologous kinetochores	precocious separations of sister chromatids; sterile tetrad	[58]
	ZmBUB1 (Maize)	Bub1-mediated phosphorylation of H2AThr133	decline of anther fertility	[59]
BUB3	ZmBUB3 (Maize)	located at the kinetochore		[59]
MAD2	MAD2 (Maize)	at centromere; relates to the distance between kinetochores		[60]
CPC proteins				
Aurora	α-Aurora β-Aurora (*Arabidopsis*)	catalytic subunit of the CPC	microsporogenesis and defects in polyploid and aneuploid offspring	[61,62]
INCENP	WYR (*Arabidopsis*)	involved in cell cycle control	defects in gametophyte cell division and development	[63]
Borealis	BORR (*Arabidopsis*)	required for proper chromosome segregation and cell division	undeveloped ovules, aborted seeds and embryonic defects	[64]

## Data Availability

The authors confirm that the data supporting the findings of this study are available within the article.

## References

[1] Earnshaw W.C. (2015). Discovering centromere proteins: From cold white hands to the A, B, C of CENPs. Nat. Rev. Mol. Cell Biol..

[2] Zafarullah M. (2017). An Investigation of Functional Dependency of CENP-C and CENH3 in *Arabidopsis thaliana*. Master’s Thesis.

[3] Heckmann S., Jankowska M., Schubert V., Kumke K., Ma W., Houben A. (2014). Alternative meiotic chromatid segregation in the holocentric plant *Luzula elegans*. Nat. Commun..

[4] Cabral G., Marques A., Schubert V., Pedrosa-Harand A., Schlögelhofer P. (2014). Chiasmatic and achiasmatic inverted meiosis of plants with holocentric chromosomes. Nat. Commun..

[5] Cao L., Li C., Li H., Wang Z., Jiang Y., Guo Y., Sun P., Chen X., Li Q., Tian H. (2022). Disruption of REC8 in Meiosis I led to watermelon seedless. Plant Sci..

[6] Watts A., Kumar V., Raipuria R.K., Bhattacharya R. (2018). In vivo haploid production in crop plants: Methods and challenges. Plant Mol. Biol. Rep..

[7] Wang K. (2020). Fixation of hybrid vigor in rice: Synthetic apomixis generated by genome editing. aBIOTECH.

[8] Xie E., Li Y., Tang D., Lv Y., Shen Y., Cheng Z. (2019). A strategy for generating rice apomixis by gene editing. J. Integr. Plant Biol..

[9] Meng D., Liu C., Chen S., Jin W. (2021). Haploid induction and its application in maize breeding. Mol. Breed..

[10] Earnshaw W.C., Rothfield N. (1985). Identification of a family of human centromere proteins using autoimmune sera from patients with scleroderma. Chromosoma.

[11] Malik H.S., Henikoff S. (2003). Phylogenomics of the nucleosome. Nat. Struct. Mol. Biol..

[12] Schubert V., Lermontova I., Schubert I. (2014). Loading of the centromeric histone H3 variant during meiosis–how does it differ from mitosis?. Chromosoma.

[13] Lermontova I., Koroleva O., Rutten T., Fuchs J., Schubert V., Moraes I., Koszegi D., Schubert I. (2011). Knockdown of CENH3 in *Arabidopsis* reduces mitotic divisions and causes sterility by disturbed meiotic chromosome segregation. Plant J..

[14] Jokelainen P.T. (1967). The ultrastructure and spatial organization of the metaphase kinetochore in mitotic rat cells. J. Ultrastruct. Res..

[15] Ravi M., Chan S.W. (2010). Haploid plants produced by centromere-mediated genome elimination. Nature.

[16] Cortes-Silva N., Ulmer J., Kiuchi T., Hsieh E., Cornilleau G., Ladid I., Dingli F., Loew D., Katsuma S., Drinnenberg I.A. (2020). CenH3-independent kinetochore assembly in Lepidoptera requires CCAN, including CENP-T. Curr. Biol..

[17] Perpelescu M., Fukagawa T. (2011). The abcs of cenps. Chromosoma.

[18] Kozgunova E., Nishina M., Goshima G. (2019). Kinetochore protein depletion underlies cytokinesis failure and somatic polyploidization in the moss *Physcomitrella patens*. eLife.

[19] Bhattacharjee S., Osman F., Feeney L., Lorenz A., Bryer C., Whitby M.C. (2013). MHF1–2/CENP-SX performs distinct roles in centromere metabolism and genetic recombination. Open Biol..

[20] Dawe R.K., Reed L.M., Yu H.-G., Muszynski M.G., Hiatt E.N. (1999). A maize homolog of mammalian CENPC is a constitutive component of the inner kinetochore. Plant Cell.

[21] Ogura Y., Shibata F., Sato H., Murata M. (2004). Characterization of a CENP-C homolog in *Arabidopsis thaliana*. Genes Genet. Syst..

[22] Tanaka K., Chang H.L., Kagami A., Watanabe Y. (2009). CENP-C functions as a scaffold for effectors with essential kinetochore functions in mitosis and meiosis. Dev. Cell.

[23] Unhavaithaya Y., Orr-Weaver T.L. (2013). Centromere proteins CENP-C and CAL1 functionally interact in meiosis for centromere clustering, pairing, and chromosome segregation. Proc. Natl. Acad. Sci. USA.

[24] Fan J., Liu Y., Zhong Y. (2021). Immunization with CENP-C causes aberrant chromosome segregation during oocyte meiosis in mice. J. Immunol. Res..

[25] Kral L.G. (2016). Possible identification of CENP-C in fish and the presence of the CENP-C motif in M18BP1 of vertebrates. F1000Research.

[26] Fellmeth J.E., McKim K.S. (2020). Meiotic CENP-C is a shepherd: Bridging the space between the centromere and the kinetochore in time and space. Essays Biochem..

[27] Gopalakrishnan S., Sullivan B.A., Trazzi S., Della Valle G., Robertson K.D. (2009). DNMT3B interacts with constitutive centromere protein CENP-C to modulate DNA methylation and the histone code at centromeric regions. Hum. Mol. Genet..

[28] Hori T., Shang W.-H., Hara M., Ariyoshi M., Arimura Y., Fujita R., Kurumizaka H., Fukagawa T. (2017). Association of M18BP1/KNL2 with CENP-A nucleosome is essential for centromere formation in non-mammalian vertebrates. Dev. Cell.

[29] Sandmann M., Talbert P., Demidov D., Kuhlmann M., Rutten T., Conrad U., Lermontova I. (2017). Targeting of *Arabidopsis* KNL2 to centromeres depends on the conserved CENPC-k motif in its C terminus. Plant Cell.

[30] Lermontova I., Kuhlmann M., Friedel S., Rutten T., Heckmann S., Sandmann M., Demidov D., Schubert V., Schubert I. (2013). *Arabidopsis* kinetochore null2 is an upstream component for centromeric histone H3 variant cenH3 deposition at centromeres. Plant Cell.

[31] Hayashi T., Fujita Y., Iwasaki O., Adachi Y., Takahashi K., Yanagida M. (2004). Mis16 and Mis18 are required for CENP-A loading and histone deacetylation at centromeres. Cell.

[32] De Rop V., Padeganeh A., Maddox P.S. (2012). CENP-A: The key player behind centromere identity, propagation, and kinetochore assembly. Chromosoma.

[33] Zuo S., Yadala R., Yang F., Talbert P., Fuchs J., Schubert V., Ahmadli U., Rutten T., Pecinka A., Lysak M.A. (2022). Recurrent plant-specific duplications of KNL2 and its conserved function as a kinetochore assembly factor. Mol. Biol. Evol..

[34] Zhang D., Martyniuk C.J., Trudeau V.L. (2006). SANTA domain: A novel conserved protein module in Eukaryota with potential involvement in chromatin regulation. Bioinformatics.

[35] Dambacher S., Deng W., Hahn M., Sadic D., Fröhlich J., Nuber A., Hoischen C., Diekmann S., Leonhardt H., Schotta G. (2012). CENP-C facilitates the recruitment of M18BP1 to centromeric chromatin. Nucleus.

[36] Stellfox M.E., Nardi I.K., Knippler C.M., Foltz D.R. (2016). Differential binding partners of the Mis18α/β YIPPEE domains regulate Mis18 complex recruitment to centromeres. Cell Rep..

[37] Fujita Y., Hayashi T., Kiyomitsu T., Toyoda Y., Kokubu A., Obuse C., Yanagida M. (2007). Priming of centromere for CENP-A recruitment by human hMis18α, hMis18β, and M18BP1. Dev. Cell.

[38] Su H., Liu Y., Wang C., Liu Y., Feng C., Sun Y., Yuan J., Birchler J.A., Han F. (2021). Knl1 participates in spindle assembly checkpoint signaling in maize. Proc. Natl. Acad. Sci. USA.

[39] Neumann P., Oliveira L.C., Jang T.S., Novak P., Koblizkova A., Schubert V., Houben A., Macas J. (2023). Disruption of the standard kinetochore in holocentric *Cuscuta* species. Proc. Natl. Acad. Sci. USA.

[40] Du Y., Dawe R.K. (2007). Maize NDC80 is a constitutive feature of the central kinetochore. Chromosome Res..

[41] Li X., Dawe R.K. (2009). Fused sister kinetochores initiate the reductional division in meiosis I. Nat. Cell Biol..

[42] Cheeseman I.M., Chappie J.S., Wilson-Kubalek E.M., Desai A. (2006). The conserved KMN network constitutes the core microtubule-binding site of the kinetochore. Cell.

[43] Petrovic A., Mosalaganti S., Keller J., Mattiuzzo M., Overlack K., Krenn V., De Antoni A., Wohlgemuth S., Cecatiello V., Pasqualato S. (2014). Modular assembly of RWD domains on the Mis12 complex underlies outer kinetochore organization. Mol. Cell.

[44] Petrovic A., Pasqualato S., Dube P., Krenn V., Santaguida S., Cittaro D., Monzani S., Massimiliano L., Keller J., Tarricone A. (2010). The MIS12 complex is a protein interaction hub for outer kinetochore assembly. J. Cell Biol..

[45] Allipra S., Anirudhan K., Shivanandan S., Raghunathan A., Maruthachalam R. (2022). The kinetochore protein NNF1 has a moonlighting role in the vegetative development of *Arabidopsis thaliana*. Plant J..

[46] Li J., Wang Y., Zou W., Jian L., Fu Y., Zhao J. (2021). AtNUF2 modulates spindle microtubule organization and chromosome segregation during mitosis. Plant J..

[47] Shin J., Jeong G., Park J.Y., Kim H., Lee I. (2018). MUN (MERISTEM UNSTRUCTURED), encoding a SPC24 homolog of NDC80 kinetochore complex, affects development through cell division in *Arabidopsis thaliana*. Plant J..

[48] Zhou J., Liu Y., Guo X., Birchler J.A., Han F., Su H. (2022). Centromeres: From chromosome biology to biotechnology applications and synthetic genomes in plants. Plant Biotechnol. J..

[49] London N., Biggins S. (2014). Signalling dynamics in the spindle checkpoint response. Nat. Rev. Mol. Cell Biol..

[50] van Hooff J.J., Tromer E., van Wijk L.M., Snel B., Kops G.J. (2017). Evolutionary dynamics of the kinetochore network in eukaryotes as revealed by comparative genomics. EMBO Rep..

[51] Sun S.-C., Kim N.-H. (2012). Spindle assembly checkpoint and its regulators in meiosis. Hum. Reprod. Update.

[52] Liu B., Lee Y.-R.J. (2022). Spindle assembly and mitosis in plants. Annu. Rev. Plant Biol..

[53] Marston A.L., Wassmann K. (2017). Multiple duties for spindle assembly checkpoint kinases in meiosis. Front. Cell Dev. Biol..

[54] Jiang H., Wang F.F., Wu Y.T., Zhou X., Huang X.Y., Zhu J., Gao J.F., Dong R.B., Cao K.M., Yang Z.N. (2009). MULTIPOLAR SPINDLE 1 (MPS1), a novel coiled-coil protein of *Arabidopsis thaliana*, is required for meiotic spindle organization. Plant J..

[55] De Muyt A., Pereira L., Vezon D., Chelysheva L., Gendrot G., Chambon A., Lainé-Choinard S., Pelletier G., Mercier R., Nogue F. (2009). A high throughput genetic screen identifies new early meiotic recombination functions in *Arabidopsis thaliana*. PLoS Genet..

[56] Xue Z., Liu C., Shi W., Miao Y., Shen Y., Tang D., Li Y., You A., Xu Y., Chong K. (2019). OsMTOPVIB is required for meiotic bipolar spindle assembly. Proc. Natl. Acad. Sci. USA.

[57] Wang C., Qu S., Zhang J., Fu M., Chen X., Liang W. (2022). OsPRD2 is essential for double-strand break formation, but not spindle assembly during rice meiosis. Front. Plant Sci..

[58] Wang M., Tang D., Luo Q., Jin Y., Shen Y., Wang K., Cheng Z. (2012). BRK1, a Bub1-related kinase, is essential for generating proper tension between homologous kinetochores at metaphase I of rice meiosis. Plant Cell.

[59] Vaur S., Cubizolles F., Plane G., Genier S., Rabitsch P.K., Gregan J., Nasmyth K., Vanoosthuyse V., Hardwick K.G., Javerzat J.-P. (2005). Control of Shugoshin function during fission-yeast meiosis. Curr. Biol..

[60] Nicklas R.B., Waters J.C., Salmon E., Ward S.C. (2001). Checkpoint signals in grasshopper meiosis are sensitive to microtubule attachment, but tension is still essential. J. Cell Sci..

[61] Weimer A.K., Demidov D., Lermontova I., Beeckman T., Van Damme D. (2016). Aurora kinases throughout plant development. Trends Plant Sci..

[62] Demidov D., Van Damme D., Geelen D., Blattner F.R., Houben A. (2005). Identification and dynamics of two classes of aurora-like kinases in *Arabidopsis* and other plants. Plant Cell.

[63] Jeyaprakash A.A., Klein U.R., Lindner D., Ebert J., Nigg E.A., Conti E. (2007). Structure of a Survivin–Borealin–INCENP core complex reveals how chromosomal passengers travel together. Cell.

[64] Kaitna S., Mendoza M., Jantsch-Plunger V., Glotzer M. (2000). Incenp and an aurora-like kinase form a complex essential for chromosome segregation and efficient completion of cytokinesis. Curr. Biol..

[65] Winey M., Goetsch L., Baum P., Byers B. (1991). MPS1 and MPS2: Novel yeast genes defining distinct steps of spindle pole body duplication. J. Cell Biol..

[66] Lauze E.Y., Stoelcker B., Luca F., Weiss E., Schutz A., Winey M. (1995). Yeast spindle pole body duplication gene MPS1 encodes an essential dual specificity protein kinase. EMBO J..

[67] Espeut J., Lara-Gonzalez P., Sassine M., Shiau A.K., Desai A., Abrieu A. (2015). Natural loss of Mps1 kinase in nematodes uncovers a role for polo-like kinase 1 in spindle checkpoint initiation. Cell Rep..

[68] Maure J.-F., Kitamura E., Tanaka T.U. (2017). Mps1 kinase promotes sister-kinetochore bi-orientation by a tension-dependent mechanism. Curr. Biol..

[69] Hewitt L., Tighe A., Santaguida S., White A.M., Jones C.D., Musacchio A., Green S., Taylor S.S. (2010). Sustained Mps1 activity is required in mitosis to recruit O-Mad2 to the Mad1–C-Mad2 core complex. J. Cell Biol..

[70] Maciejowski J., George K.A., Terret M.-E., Zhang C., Shokat K.M., Jallepalli P.V. (2010). Mps1 directs the assembly of Cdc20 inhibitory complexes during interphase and mitosis to control M phase timing and spindle checkpoint signaling. J. Cell Biol..

[71] Santaguida S., Tighe A., D’Alise A.M., Taylor S.S., Musacchio A. (2010). Dissecting the role of MPS1 in chromosome biorientation and the spindle checkpoint through the small molecule inhibitor reversine. J. Cell Biol..

[72] Tighe A., Staples O., Taylor S. (2008). Mps1 kinase activity restrains anaphase during an unperturbed mitosis and targets Mad2 to kinetochores. J. Cell Biol..

[73] Benzi G., Camasses A., Atsunori Y., Katou Y., Shirahige K., Piatti S. (2020). A common molecular mechanism underlies the role of Mps1 in chromosome biorientation and the spindle assembly checkpoint. EMBO Rep..

[74] London N., Ceto S., Ranish J.A., Biggins S. (2012). Phosphoregulation of Spc105 by Mps1 and PP1 regulates Bub1 localization to kinetochores. Curr. Biol..

[75] Ji Z., Gao H., Jia L., Li B., Yu H. (2017). A sequential multi-target Mps1 phosphorylation cascade promotes spindle checkpoint signaling. eLife.

[76] Tipton A.R., Ji W., Sturt-Gillespie B., Bekier M.E., Wang K., Taylor W.R., Liu S.-T. (2013). Monopolar spindle 1 (MPS1) kinase promotes production of closed MAD2 (C-MAD2) conformer and assembly of the mitotic checkpoint complex. J. Biol. Chem..

[77] Straight P.D., Giddings T.H., Winey M. (2000). Mps1p regulates meiotic spindle pole body duplication in addition to having novel roles during sporulation. Mol. Biol. Cell..

[78] Gilliland W.D., Wayson S.M., Hawley R.S. (2005). The meiotic defects of mutants in the *Drosophila* mps1 gene reveal a critical role of Mps1 in the segregation of achiasmate homologs. Curr. Biol..

[79] Gilliland W.D., Hughes S.E., Cotitta J.L., Takeo S., Xiang Y., Hawley R.S. (2007). The multiple roles of Mps1 in *Drosophila* female meiosis. PLoS Genet..

[80] Hached K., Xie S.Z., Buffin E., Cladière D., Rachez C., Sacras M., Sorger P.K., Wassmann K. (2011). Mps1 at kinetochores is essential for female mouse meiosis I. Development.

[81] Paganelli L., Caillaud M.C., Quentin M., Damiani I., Govetto B., Lecomte P., Karpov P.A., Abad P., Chabouté M.E., Favery B. (2015). Retracted: Three BUB 1 and BUBR 1/MAD 3-related spindle assembly checkpoint proteins are required for accurate mitosis in *Arabidopsis*. New Phytol..

[82] Su H., Liu Y., Dong Q., Feng C., Zhang J., Liu Y., Birchler J.A., Han F. (2017). Dynamic location changes of Bub1-phosphorylated-H2AThr133 with CENH3 nucleosome in maize centromeric regions. New Phytol..

[83] Miyazaki S., Kim J., Yamagishi Y., Ishiguro T., Okada Y., Tanno Y., Sakuno T., Watanabe Y. (2017). Meikin-associated polo-like kinase specifies Bub1 distribution in meiosis I. Genes Cells.

[84] Yamaguchi S., Decottignies A., Nurse P. (2003). Function of Cdc2p-dependent Bub1p phosphorylation and Bub1p kinase activity in the mitotic and meiotic spindle checkpoint. EMBO J..

[85] Li M., Li S., Yuan J., Wang Z.-B., Sun S.-C., Schatten H., Sun Q.-Y. (2009). Bub3 is a spindle assembly checkpoint protein regulating chromosome segregation during mouse oocyte meiosis. PLoS ONE.

[86] Fraschini R., Beretta A., Sironi L., Musacchio A., Lucchini G., Piatti S. (2001). Bub3 interaction with Mad2, Mad3 and Cdc20 is mediated by WD40 repeats and does not require intact kinetochores. EMBO J..

[87] Lermontova I., Fuchs J., Schubert I. (2008). The *Arabidopsis* checkpoint protein Bub3. 1 is essential for gametophyte development. Front. Biosci..

[88] Komaki S., Schnittger A. (2017). The spindle assembly checkpoint in *Arabidopsis* is rapidly shut off during severe stress. Dev. Cell.

[89] Zhang H., Deng X., Sun B., Lee Van S., Kang Z., Lin H., Lee Y.-R.J., Liu B. (2018). Role of the BUB3 protein in phragmoplast microtubule reorganization during cytokinesis. Nat. Plants.

[90] Rancati G., Crispo V., Lucchini G., Piatti S. (2005). Mad3/BubR1 phosphorylation during spindle checkpoint activation depends on both Polo and Aurora kinases in budding yeast. Cell Cycle.

[91] Pérez-Mongiovi D., Malmanche N., Bousbaa H., Sunkel C. (2005). Maternal expression of the checkpoint protein BubR1 is required for synchrony of syncytial nuclear divisions and polar body arrest in *Drosophila melanogaster*. Development.

[92] Wei L., Liang X.-W., Zhang Q.-H., Li M., Yuan J., Li S., Sun S.-C., Ouyang Y.-C., Schatten H., Sun Q.-Y. (2010). BubR1 is a spindle assembly checkpoint protein regulating meiotic cell cycle progression of mouse oocyte. Cell Cycle.

[93] Komaki S., Schnittger A. (2016). The spindle checkpoint in plants—A green variation over a conserved theme?. Curr. Opin. Plant Biol..

[94] Parra M.T., Gomez R., Viera A., Llano E., Pendas A.M., Rufas J.S., Suja J.A. (2009). Sequential assembly of centromeric proteins in male mouse meiosis. PLoS Genet..

[95] Jeganathan K.B., Van Deursen J. (2006). Differential mitotic checkpoint protein requirements in somatic and germ cells. Biochem. Soc. Trans..

[96] Heng-Yu F. (2010). BubR1, a spindle assembly checkpoint protein in mammalian oocyte meiosis. Cell Cycle.

[97] Caillaud M.-C., Paganelli L., Lecomte P., Deslandes L., Quentin M., Pecrix Y., Le Bris M., Marfaing N., Abad P., Favery B. (2009). Spindle assembly checkpoint protein dynamics reveal conserved and unsuspected roles in plant cell division. PLoS ONE.

[98] Kimbara J., Endo T.R., Nasuda S. (2004). Characterization of the genes encoding for MAD2 homologues in wheat. Chromosome Res..

[99] Chen R.-H., Brady D.M., Smith D., Murray A.W., Hardwick K.G. (1999). The spindle checkpoint of budding yeast depends on a tight complex between the Mad1 and Mad2 proteins. Mol. Biol. Cell..

[100] Cheslock P.S., Kemp B.J., Boumil R.M., Dawson D.S. (2005). The roles of MAD1, MAD2 and MAD3 in meiotic progression and the segregation of nonexchange chromosomes. Nat. Genet..

[101] Kitagawa R., Rose A.M. (1999). Components of the spindle-assembly checkpoint are essential in *Caenorhabditis elegans*. Nat. Cell Biol..

[102] Zhang D., Li M., Ma W., Hou Y., Li Y.-H., Li S.-W., Sun Q.-Y., Wang W.-H. (2005). Localization of mitotic arrest deficient 1 (MAD1) in mouse oocytes during the first meiosis and its functions as a spindle checkpoint protein. Biol. Reprod..

[103] Ding D., Muthuswamy S., Meier I. (2012). Functional interaction between the *Arabidopsis* orthologs of spindle assembly checkpoint proteins MAD1 and MAD2 and the nucleoporin NUA. Plant Mol. Biol..

[104] Bao Z., Zhang N., Hua J. (2014). Endopolyploidization and flowering time are antagonistically regulated by checkpoint component MAD1 and immunity modulator MOS1. Nat. Commun..

[105] Kallio M., Eriksson J.E., Gorbsky G.J. (2000). Differences in spindle association of the mitotic checkpoint protein Mad2 in mammalian spermatogenesis and oogenesis. Dev. Biol..

[106] Zhang D., Ma W., Li Y.-H., Hou Y., Li S.-W., Meng X.-Q., Sun X.-F., Sun Q.-Y., Wang W.-H. (2004). Intra-oocyte localization of MAD2 and its relationship with kinetochores, microtubules, and chromosomes in rat oocytes during meiosis. Biol. Reprod..

[107] Yu H.-G., Muszynski M.G., Kelly Dawe R. (1999). The maize homologue of the cell cycle checkpoint protein MAD2 reveals kinetochore substructure and contrasting mitotic and meiotic localization patterns. J. Cell Biol..

[108] Niault T., Hached K., Sotillo R., Sorger P.K., Maro B., Benezra R., Wassmann K. (2007). Changing Mad2 levels affects chromosome segregation and spindle assembly checkpoint control in female mouse meiosis I. PLoS ONE.

[109] Wang J.Y., Lei Z.L., Nan C.L., Yin S., Liu J., Hou Y., Li Y.L., Chen D.Y., Sun Q.Y. (2007). RNA interference as a tool to study the function of MAD2 in mouse oocyte meiotic maturation. Mol. Reprod. Dev..

[110] Yamamoto A., Kitamura K., Hihara D., Hirose Y., Katsuyama S., Hiraoka Y. (2008). Spindle checkpoint activation at meiosis I advances anaphase II onset via meiosis-specific APC/C regulation. J. Cell Biol..

[111] Ruchaud S., Carmena M., Earnshaw W.C. (2007). Chromosomal passengers: Conducting cell division. Nat. Rev. Mol. Cell Biol..

[112] Vader G., Cruijsen C.W., Van Harn T., Vromans M.J., Medema R.H., Lens S.M. (2007). The chromosomal passenger complex controls spindle checkpoint function independent from its role in correcting microtubule–kinetochore interactions. Mol. Biol. Cell.

[113] Muñoz-Barrera M., Monje-Casas F. (2014). Increased Aurora B activity causes continuous disruption of kinetochore–microtubule attachments and spindle instability. Proc. Natl. Acad. Sci. USA.

[114] Landino J., Ohi R. (2016). The timing of midzone stabilization during cytokinesis depends on myosin II activity and an interaction between INCENP and actin. Curr. Biol..

[115] Kitagawa M., Lee S.H. (2015). The chromosomal passenger complex (CPC) as a key orchestrator of orderly mitotic exit and cytokinesis. Front. Cell Dev. Biol..

[116] Giet R., Prigent C. (1999). Aurora/Ipl1p-related kinases, a new oncogenic family of mitotic serine-threonine kinases. J. Cell Sci..

[117] Petersen J., Paris J., Willer M., Philippe M., Hagan I.M. (2001). The *S. pombe* aurora-related kinase Ark1 associates with mitotic structures in a stage dependent manner and is required for chromosome segregation. J. Cell Sci..

[118] Ducat D., Zheng Y. (2004). Aurora kinases in spindle assembly and chromosome segregation. Exp. Cell Res..

[119] Goldenson B., Crispino J.D. (2015). The aurora kinases in cell cycle and leukemia. Oncogene.

[120] Carmena M., Earnshaw W.C. (2003). The cellular geography of aurora kinases. Nat. Rev. Mol. Cell Biol..

[121] Yu H.-G., Koshland D. (2007). The Aurora kinase Ipl1 maintains the centromeric localization of PP2A to protect cohesin during meiosis. J. Cell Biol..

[122] Monje-Casas F., Prabhu V.R., Lee B.H., Boselli M., Amon A. (2007). Kinetochore orientation during meiosis is controlled by Aurora B and the monopolin complex. Cell.

[123] Balboula A.Z., Schindler K. (2014). Selective disruption of aurora C kinase reveals distinct functions from aurora B kinase during meiosis in mouse oocytes. PLoS Genet..

[124] Tang C.-J.C., Lin C.-Y., Tang T.K. (2006). Dynamic localization and functional implications of Aurora-C kinase during male mouse meiosis. Dev. Biol..

[125] Demidov D., Lermontova I., Weiss O., Fuchs J., Rutten T., Kumke K., Sharbel T.F., Van Damme D., De Storme N., Geelen D. (2014). Altered expression of Aurora kinases in *Arabidopsis* results in aneu-and polyploidization. Plant J..

[126] Niu B., Wang L., Zhang L., Ren D., Ren R., Copenhaver G.P., Ma H., Wang Y. (2015). *Arabidopsis* cell division cycle 20.1 is required for normal meiotic spindle assembly and chromosome segregation. Plant Cell.

[127] Zamariola L., Tiang C.L., De Storme N., Pawlowski W., Geelen D. (2014). Chromosome segregation in plant meiosis. Front. Plant Sci..

[128] Carmena M., Wheelock M., Funabiki H., Earnshaw W.C. (2012). The chromosomal passenger complex (CPC): From easy rider to the godfather of mitosis. Nat. Rev. Mol. Cell Biol..

[129] Komaki S., Takeuchi H., Hamamura Y., Heese M., Hashimoto T., Schnittger A. (2020). Functional analysis of the plant chromosomal passenger complex. Plant Physiol..

[130] Kirioukhova O., Johnston A.J., Kleen D., Kägi C., Baskar R., Moore J.M., Bäumlein H., Groß-Hardt R., Grossniklaus U. (2011). Female gametophytic cell specification and seed development require the function of the putative *Arabidopsis* INCENP ortholog WYRD. Development.

[131] Gassmann R., Carvalho A., Henzing A.J., Ruchaud S., Hudson D.F., Honda R., Nigg E.A., Gerloff D.L., Earnshaw W.C. (2004). Borealin: A novel chromosomal passenger required for stability of the bipolar mitotic spindle. J. Cell Biol..

[132] Komaki S., Tromer E.C., De Jaeger G., De Winne N., Heese M., Schnittger A. (2022). Molecular convergence by differential domain acquisition is a hallmark of chromosomal passenger complex evolution. Proc. Natl. Acad. Sci. USA.

[133] Sun S.-C., Wei L., Li M., Lin S.-L., Xu B.-Z., Liang X.-W., Kim N.-H., Schatten H., Lu S.-S., Sun Q.-Y. (2009). Perturbation of survivin expression affects chromosome alignment and spindle checkpoint in mouse oocyte meiotic maturation. Cell Cycle.

[134] Wang K., Jiang G.-J., Wei L., Liang X.-W., Miao D.-Q., Sun S.-C., Guo L., Wang Z.-B., Lu S.-S. (2011). Survivin is a critical regulator of spindle organization and chromosome segregation during rat oocyte meiotic maturation. Zygote.

[135] Chelysheva L., Diallo S., Vezon D., Gendrot G., Vrielynck N., Belcram K., Rocques N., Márquez-Lema A., Bhatt A.M., Horlow C. (2005). AtREC8 and AtSCC3 are essential to the monopolar orientation of the kinetochores during meiosis. J. Cell Sci..

[136] Cuacos M., H. Franklin F.C., Heckmann S. (2015). Atypical centromeres in plants—What they can tell us. Front. Plant Sci..

[137] Nasmyth K., Haering C.H. (2005). The structure and function of SMC and kleisin complexes. Annu. Rev. Biochem..

[138] Haering C.H., Löwe J., Hochwagen A., Nasmyth K. (2002). Molecular architecture of SMC proteins and the yeast cohesin complex. Mol. Cell.

[139] Hirano T. (2005). SMC proteins and chromosome mechanics: From bacteria to humans. Philos. Trans. R. Soc. Lond. B Biol. Sci..

[140] Schubert V. (2009). SMC proteins and their multiple functions in higher plants. Cytogenet. Genome Res..

[141] Cai X., Dong F., Edelmann R.E., Makaroff C.A. (2003). The *Arabidopsis* SYN1 cohesin protein is required for sister chromatid arm cohesion and homologous chromosome pairing. J. Cell Sci..

[142] Kudo N.R., Anger M., Peters A.H., Stemmann O., Theussl H.-C., Helmhart W., Kudo H., Heyting C., Nasmyth K. (2009). Role of cleavage by separase of the Rec8 kleisin subunit of cohesin during mammalian meiosis I. J. Cell Sci..

[143] Watanabe Y., Nurse P. (1999). Cohesin Rec8 is required for reductional chromosome segregation at meiosis. Nature.

[144] Sakuno T., Hiraoka Y. (2022). Rec8 cohesin: A structural platform for shaping the meiotic chromosomes. Genes.

[145] Kudo N.R., Wassmann K., Anger M., Schuh M., Wirth K.G., Xu H., Helmhart W., Kudo H., Mckay M., Maro B. (2006). Resolution of chiasmata in oocytes requires separase-mediated proteolysis. Cell.

[146] Martinez-Perez E., Schvarzstein M., Barroso C., Lightfoot J., Dernburg A.F., Villeneuve A.M. (2008). Crossovers trigger a remodeling of meiotic chromosome axis composition that is linked to two-step loss of sister chromatid cohesion. Genes Dev..

[147] Xu H., Beasley M.D., Warren W.D., van der Horst G.T., McKay M.J. (2005). Absence of mouse REC8 cohesin promotes synapsis of sister chromatids in meiosis. Dev. Cell.

[148] Doll E., Molnar M., Cuanoud G., Octobre G., Latypov V., Ludin K., Kohli J.R. (2008). Cohesin and recombination proteins influence the G1-to-S transition in azygotic meiosis in *Schizosaccharomyces pombe*. Genetics.

[149] Peirson B.N., Bowling S.E., Makaroff C.A. (1997). A defect in synapsis causes male sterility in a T-DNA-tagged *Arabidopsis thaliana* mutant. Plant J..

[150] Lam W.S., Yang X., Makaroff C.A. (2005). Characterization of *Arabidopsis thaliana* SMC1 and SMC3: Evidence that AtSMC3 may function beyond chromosome cohesion. J. Cell Sci..

[151] Lhuissier F.G., Offenberg H.H., Wittich P.E., Vischer N.O., Heyting C. (2007). The mismatch repair protein MLH1 marks a subset of strongly interfering crossovers in tomato. Plant Cell.

[152] Mercier R., Grelon M. (2008). Meiosis in plants: Ten years of gene discovery. Cytogenet. Genome Res..

[153] Mercier R., Mézard C., Jenczewski E., Macaisne N., Grelon M. (2015). The molecular biology of meiosis in plants. Annu. Rev. Plant Biol..

[154] Hamant O., Ma H., Cande W.Z. (2006). Genetics of meiotic prophase I in plants. Annu. Rev. Plant Biol..

[155] Shonn M.A., McCarroll R., Murray A.W. (2002). Spo13 protects meiotic cohesin at centromeres in meiosis I. Genes Dev..

[156] Yokobayashi S., Watanabe Y. (2005). The kinetochore protein Moa1 enables cohesion-mediated monopolar attachment at meiosis I. Cell.

[157] Kim J., Ishiguro K.-i., Nambu A., Akiyoshi B., Yokobayashi S., Kagami A., Ishiguro T., Pendas A.M., Takeda N., Sakakibara Y. (2015). Meikin is a conserved regulator of meiosis-I-specific kinetochore function. Nature.

[158] Bonner A.M., Hughes S.E., Hawley R.S. (2020). Regulation of polo kinase by matrimony is required for cohesin maintenance during *Drosophila melanogaster* female meiosis. Curr. Biol..

[159] Yokobayashi S., Yamamoto M., Watanabe Y. (2003). Cohesins determine the attachment manner of kinetochores to spindle microtubules at meiosis I in fission yeast. Mol. Cell. Biol..

[160] Ferrandiz N., Barroso C., Telecan O., Shao N., Kim H.-M., Testori S., Faull P., Cutillas P., Snijders A.P., Colaiácovo M.P. (2018). Spatiotemporal regulation of Aurora B recruitment ensures release of cohesion during *C. elegans* oocyte meiosis. Nat. Commun..

[161] McKee B.D., Yan R., Tsai J.-H. (2012). Meiosis in male Drosophila. Spermatogenesis.

[162] Yu H.-G., Dawe R.K. (2000). Functional redundancy in the maize meiotic kinetochore. J. Cell Biol..

[163] Shao T., Tang D., Wang K., Wang M., Che L., Qin B., Yu H., Li M., Gu M., Cheng Z. (2011). OsREC8 is essential for chromatid cohesion and metaphase I monopolar orientation in rice meiosis. Plant Physiol..

[164] Bai X., Peirson B.N., Dong F., Xue C., Makaroff C.A. (1999). Isolation and characterization of SYN1, a RAD21-like gene essential for meiosis in *Arabidopsis*. Plant Cell.

[165] Bhatt A.M., Lister C., Page T., Fransz P., Findlay K., Jones G.H., Dickinson H.G., Dean C. (1999). The DIF1 gene of *Arabidopsis* is required for meiotic chromosome segregation and belongs to the REC8/RAD21 cohesin gene family. Plant J..

[166] Zhang L., Tao J., Wang S., Chong K., Wang T. (2006). The rice OsRad21-4, an orthologue of yeast Rec8 protein, is required for efficient meiosis. Plant Mol. Biol..

[167] Moens P.B., Kolas N.K., Tarsounas M., Marcon E., Cohen P.E., Spyropoulos B. (2002). The time course and chromosomal localization of recombination-related proteins at meiosis in the mouse are compatible with models that can resolve the early DNA-DNA interactions without reciprocal recombination. J. Cell Sci..

[168] Watanabe Y. (2004). Modifying sister chromatid cohesion for meiosis. J. Cell Sci..

[169] Altschul S.F., Gish W., Miller W., Myers E.W., Lipman D.J. (1990). Basic local alignment search tool. J. Mol. Biol..

[170] Kerrebrock A.W., Moore D.P., Wu J.S., Orr-Weaver T.L. (1995). Mei-S332, a *Drosophila* protein required for sister-chromatid cohesion, can localize to meiotic centromere regions. Cell.

[171] Yao Y., Dai W. (2012). Shugoshins function as a guardian for chromosomal stability in nuclear division. Cell Cycle.

[172] Katis V.L., Galova M., Rabitsch K.P., Gregan J., Nasmyth K. (2004). Maintenance of cohesin at centromeres after meiosis I in budding yeast requires a kinetochore-associated protein related to MEI-S332. Curr. Biol..

[173] Kitajima T.S., Kawashima S.A., Watanabe Y. (2004). The conserved kinetochore protein shugoshin protects centromeric cohesion during meiosis. Nature.

[174] Marston A.L., Tham W.-H., Shah H., Amon A. (2004). A genome-wide screen identifies genes required for centromeric cohesion. Science.

[175] Rabitsch K.P., Gregan J., Schleiffer A., Javerzat J.-P., Eisenhaber F., Nasmyth K. (2004). Two fission yeast homologs of *Drosophila* Mei-S332 are required for chromosome segregation during meiosis I and II. Curr. Biol..

[176] Hamant O., Golubovskaya I., Meeley R., Fiume E., Timofejeva L., Schleiffer A., Nasmyth K., Cande W.Z. (2005). A REC8-dependent plant Shugoshin is required for maintenance of centromeric cohesion during meiosis and has no mitotic functions. Curr. Biol..

[177] Gómez R., Valdeolmillos A., Parra M.T., Viera A., Carreiro C., Roncal F., Rufas J.S., Barbero J.L., Suja J.A. (2007). Mammalian SGO2 appears at the inner centromere domain and redistributes depending on tension across centromeres during meiosis II and mitosis. EMBO Rep..

[178] Lee J., Kitajima T.S., Tanno Y., Yoshida K., Morita T., Miyano T., Miyake M., Watanabe Y. (2008). Unified mode of centromeric protection by shugoshin in mammalian oocytes and somatic cells. Nat. Cell Biol..

[179] Xu Z., Cetin B., Anger M., Cho U.S., Helmhart W., Nasmyth K., Xu W. (2009). Structure and function of the PP2A-shugoshin interaction. Mol. Cell.

[180] Macy B., Wang M., Yu H.-G. (2009). The many faces of shugoshin, the “guardian spirit,” in chromosome segregation. Cell Cycle.

[181] Tanno Y., Kitajima T.S., Honda T., Ando Y., Ishiguro K.-I., Watanabe Y. (2010). Phosphorylation of mammalian Sgo2 by Aurora B recruits PP2A and MCAK to centromeres. Genes Dev..

[182] Clift D., Marston A. (2011). The role of shugoshin in meiotic chromosome segregation. Cytogenet. Genome Res..

[183] Zamariola L., De Storme N., Tiang C., Armstrong S., Franklin F., Geelen D. (2013). SGO1 but not SGO2 is required for maintenance of centromere cohesion in *Arabidopsis thaliana* meiosis. Plant Reprod..

[184] Zamariola L., De Storme N., Vannerum K., Vandepoele K., Armstrong S.J., Franklin F.C.H., Geelen D. (2014). SHUGOSHIN s and PATRONUS protect meiotic centromere cohesion in *Arabidopsis thaliana*. Plant J..

[185] Cromer L., Jolivet S., Horlow C., Chelysheva L., Heyman J., De Jaeger G., Koncz C., De Veylder L., Mercier R. (2013). Centromeric cohesion is protected twice at meiosis, by SHUGOSHINs at anaphase I and by PATRONUS at interkinesis. Curr. Biol..

[186] Wang M., Tang D., Wang K., Shen Y., Qin B., Miao C., Li M., Cheng Z. (2011). OsSGO1 maintains synaptonemal complex stabilization in addition to protecting centromeric cohesion during rice meiosis. Plant J..

[187] Mahjoubi H., Tamari Y., Takeda S., Bouchabké-Coussa O., Hanin M., Herzog E., Schmit A.-C., Chabouté M.-E., Ebel C. (2018). The wheat TdRL1 is the functional homolog of the rice RSS1 and promotes plant salt stress tolerance. Plant Cell Rep..

[188] Cromer L., Jolivet S., Singh D.K., Berthier F., De Winne N., De Jaeger G., Komaki S., Prusicki M.A., Schnittger A., Guérois R. (2019). Patronus is the elusive plant securin, preventing chromosome separation by antagonizing separase. Proc. Natl. Acad. Sci. USA.

[189] Ogawa D., Abe K., Miyao A., Kojima M., Sakakibara H., Mizutani M., Morita H., Toda Y., Hobo T., Sato Y. (2011). RSS1 regulates the cell cycle and maintains meristematic activity under stress conditions in rice. Nat. Com..

[190] Ravi M., Bondada R. (2016). Genome elimination by tailswap CenH3: In vivo haploid production in *Arabidopsis thaliana*. Chromosome and Genomic Engineering in Plants: Methods and Protocols.

[191] Marimuthu M.P., Jolivet S., Ravi M., Pereira L., Davda J.N., Cromer L., Wang L., Nogue F., Chan W.L., Siddiqi I. (2011). Synthetic clonal reproduction through seeds. Science.

[192] Wang N., Gent J.I., Dawe R.K. (2021). Haploid induction by a maize *cenh3* null mutant. Sci. Adv..

[193] Lv J., Yu K., Wei J., Gui H., Liu C., Liang D., Wang Y., Zhou H., Carlin R., Rich R. (2020). Generation of paternal haploids in wheat by genome editing of the centromeric histone CENH3. Nat. Biotechnol.

[194] Sanei M., Pickering R., Kumke K., Nasuda S., Houben A. (2011). Loss of centromeric histone H3 (CENH3) from centromeres precedes uniparental chromosome elimination in interspecific barley hybrids. Proc. Natl. Acad. Sci. USA.

